# Evaluating the impact of land use and land cover changes on sediment yield dynamics in the upper Awash basin, Ethiopia the case of Koka reservoir

**DOI:** 10.1016/j.heliyon.2023.e23049

**Published:** 2023-11-29

**Authors:** Bayu Geta Bihonegn, Admasu Gebeyehu Awoke

**Affiliations:** a Kombolcha Institute of Technology, Wollo University, Kombolcha, P.O.box. 208, Ethiopia; b Department of Civil and Environmental Engineering, Addis Ababa University, Addis Ababa Institute of Technology, Addis Ababa, Ethiopia

**Keywords:** Hydrological response, Koka dam, Land use change, QSWATPLUS model

## Abstract

Land Use and Land Cover changes (LULC) are the driving forces to change the hydrological response of the watershed. In this study, the Quantum Geography Information System Interference Soil and Water Assessment Tool Plus (QSWAT-PLUS) model was applied to evaluate the effects of LULC on sediment load at the Upper Awash River Basin (UARB) which are causing sedimentation problems in Koka reservoir. The LULC data for 2005, 2010, and 2015 were obtained from historical satellite images using Earth Resources Observation and Science (ERDAS) 2014. The classification of LULC changes showed that the agricultural practice, and the settlement land both increased by 6.7 % and 6.3 %, respectively. In contrast, the forest area, woodland, shrubland, and water bodies decreased by 5.47 %,0.93 %,0.96 %, and 1.34 % from 2000 to 2015 respectively. The model evaluation results were satisfactory for the three LULC scenarios. The average annual surface runoff volume for the 2005 LULC data was 182.2 mm, which increased to 193.29 mm in 2010 and 205.3 mm in 2015. Similarly, the average annual sediment yield that would enter to the Koka reservoir under the 2005, 2010, and 2015 LULC scenarios were 26.03 t/ha/yr, 26.34 t/ha/yr, and 28.33 t/ha/yr respectively. In general, streamflow, surface runoff, and sediment output increased by 4.55 %, 12.68 %, and 8.84 %, respectively due to the rapid change of LULC from 2000 to 2015. Temporarily, the sediment load at the upstream side of the Koka Dam watershed was 60.8 % during the wet season. The southwest direction of the watershed was identified as the primary erosion-prone area. Based on the simulation results, the filter strip, contour, and terraces reduced the watershed sediment yield by up to 60 %, 65 %, and 80 %, respectively. Therefore, the selected best management practices are highly effective in reducing silt along the entire upstream side of the Koka Dam watershed.

## 1 Introduction

Land use and land cover changes have a great impact on global environmental dynamics. It significantly affected the hydrological response, the hydrological cycle, and climate processes [1,2], and [3]. One of the effects of altering land use and land cover changes is soil erosion, which causes more silt to enter into dam reservoirs, reducing their capacity and endangering their performance [4]. In the World, many reservoirs have lost one-half of a percent of their storage capacity per annum due to sedimentation problems which is causing serious problems for hydropower, irrigation, water supply, and flood control [5].

In developing countries like Ethiopia, the highland areas are categorized as a region with high rates of soil erosion and land degradation problems [6,7], and [8]. The soil erosion and land degradation problems of the highland areas increased due to rapid LULC change. The rapid LULC changes are the results of expanded agricultural land, fast population growth, deforestation, and poor afforestation practices in the sloping area which are the main factors to accelerate soil erosion and land degradation in Ethiopia resulting in reservoir sedimentation problems [4], and [5]. Ethiopia loses 1.5 billion tons of topsoil losses in every year. It shows that soil erosion is a series issue in Ethiopia [3]. As a result, some reservoirs had sediment deposition issues and reduced their functions. Good examples of power generation and water supply reservoirs that are affected by sediment deposition problems are Koka [9], Gilgel Gibe I [10], MelkaWakena [11], Angreb [12], and Legedadi [9]. Moreover, in downstream countries like Egypt and Sudan, infrastructures such as Aswan, Rossier, and other storage reservoirs and irrigation canals have experienced serious sedimentation problems due to excessive sediment loads that originate from Ethiopian highlands [13], and [14].

Particularly, the UARB is severely affected by poor watershed management problems due to dense population, expansion of urbanization, poor forestation, deforestation, and overgrazing, resulting in perceptible loss of soil fertility, and rapid degradation of natural resources [[15], [16], [17]], and [18].

The result of soil erosion and sediment transportation into some dam reservoirs particularly in this area accelerated the sediment deposition and reduced the reservoir capacity resulting in difficulty to use the reservoirs for hydropower, flood control, irrigation, and water supply. For example, the Koka Dam reservoir lost 17 million cubic meters of storage capacity per year. Due to this, the generated power capacity of the dam reservoir has reduced in 2014. In addition to this, Awash Melkessa reservoir stopped its service due to heavy siltation problems and the Methara sugar factory was also challenging due to the high siltation of the canal and difficulty to irrigate the sugarcane plants [19].

Land use/land cover, rainfall, topography, soil properties, and geological formation are some of the many variables that affect the hydrological processes. Among such, the primary cause of the hydrologic process in watersheds is human-induced LULC change [20]. Alterations of LULC dynamics have been a major source of soil erosion and sediment yield in recent times [21,22]. The significant change in LULC is a cause of surface runoff and sediment yield variation at seasonal and spatial scales. To understand this, the change of Angerb watershed LULC from 1985 to 2011 is an explanatory example. This watershed changed the land use type from forest plantations to agricultural practices between 1985 and 2011 periods. As a result, the runoff was increased by 39 % [23]. In the Tekeze Dam watershed, the LULC type has changed from grass and bare land to agricultural land. Because of this, the average annual stream flow has increased by 6.02 %, while the amount of sediment yield increased by 17.39 % during the time interval from 1986 to 2008 [1].

Many researchers have investigated the impact of LULC change on sediment yield dynamics in the UARB. According to their findings, the LULC changes in Ethiopia's highlands are a significant issue that endangers natural ecosystems [17]. Therefore, understanding the effects of LULC change in the watershed is essential for planning and managing land use and water resources [24]. According to Shawl and Chakma's findings in the upper Awash watershed, LULC changed dramatically from shrubland to crop area [15]. This result showed how much the agricultural practice in this area has expanded from 1972 to 2014. This led to an increase in the annual sediment production in the Koka Dam reservoir. According to Ref. [25], the average annual sediment product entering the Koka Dam reservoir is 21.43 t/ha/yr. 106,352.59 tons of average annual sediment output entered to the Merti intake structure, which was built in the Awash River [26]. The sediment yield of the UARB varies from time to time. During the wet season, 70.8 % of the yearly sediment yield occurs in middle Awash catchments [27]. Even though the issue of evaluating the effects of LULC changes on sediment yield dynamics in the UARB has been the subject of numerous studies, it is necessary to urgently and seriously assess the impact of LULC changes on sediment load at Koka dam reservoirs to extend reservoir life through the application of practical land use and watershed management techniques. Furthermore, most studies were limited to small watersheds and landscapes, and calibration and validation were carried out at the Hombole gauge station, which had a coverage area of around 7000 km^2^. But, the calibration and validation for this study were performed at the upstream side of the dam which covered 10,000 km^2^ watershed area. Unlikely the previous studies, in this study, the new version of the QSWATPLUS model was used to evaluate the effects of land use change on sediment variation by using different years of LULC data and to evaluate the best watershed management strategies.

The Awash River basin is the backbone of economic development in Ethiopia. Large sugar and hydro-power plants have been found in this basin, and they are vital to the country's economy. However, the UARB is currently suffering from land degradation problem caused by urbanization, dense population, agricultural development, and poor watershed management [24]. On top of that, the basin is environmentally vulnerable [28,29], and [18] and the river is characterized by high flash floods which carry a high amount of silt that significantly affects the capacity of the Koka reservoir. Due to sediment deposition in the reservoir, Koka's power generation capability was reduced from the expected power output. In general, most reservoirs have major sedimentation problems [30]. Therefore, evaluating the impact of LULC change on sediment yield dynamics in the upper Awash River basin is critical for providing useful information to water resource and land use planners in developing subbasin management strategies and reducing sediment deposition at Koka Reservoir using effective watershed management strategies.

Various physical-based soil erosion models have been developed worldwide to anticipate soil loss and sediment load and to assess the impact of LULC change on watersheds [31]. They also help to identify the erosion-prone area and to determine the optimum management approach for the erosion-prone area in a timely and cost-effective manner. For instance, Annualized Agricultural Non-Point Source (AnnAGNPS) [32], SWAT [33], Water Erosion Prediction Project (WEPP) [34], QSWATPlus [35,36], and European soil erosion model (EUROSEM) [37] such physical models have been used in the recent years to evaluate the impact of LULC change on soil erosion and its control mechanism. Among those models, the AnnAGNPS model requires extensive data input and no mass balance equation [38], the WEPP model also requires a large number of input data and parameters and cannot accurately simulate the process occurring in permanent channels and streams, EUROSED is relying on single storm and suitable for small catchments [37]. Whereas the SWAT model requires relatively small input data and is used to simulate long-term hydrologic, soil erosion, and sediment yield in large and complex watersheds using daily time steps. It is the most applicable model worldwide to estimate sediment yield on a daily and monthly basis and to predict long-term impact land use activities in complicated and big watersheds [39], and [40]. It was applicable for predicting sediment load, identifying the erosion area, and evaluating the best management strategies in Ethiopia's watersheds under different scenarios [41,42], and [43]. The fundamental issue of SWAT model over the other hydrological models is its capacity to predict sediment load if the observed streamflow and sediment data are not sufficient and unreliable in the watershed [44]. Therefore, the QSWATPLUS model was used in this study to assess the impacts of LULC variations on sediment load in the Koka reservoir. It is the upgraded version of the SWAT model, a time-continuous semi-distributed model that works on the principle of hydrologic response units. The uniqueness of the QSWAT plus model from the previous version added the landscape units beside to sub-catchment. The importance of using the QSWAT plus model over other physical models is to understand the basic catchment characteristics when the data are not accessible and to evaluate the long-term effects of LULC change on the hydrological response that are difficult to simulate [45].

In this study, it was hypothesized that major changes in the LULC in the UARB are related to human activities that provide for the needs of the fast-rising population in terms of food security by utilizing improper and ill-planned natural resource management practices.

The particular aims of this study are as follows: i) to assess the impact of land use land cover changes on sediment yield dynamics in the upper Koka Dam watershed using the 2000,2005,2010 and 2015 LULC maps ii) to estimate the mean annual sediment yield loading to the Koka reservoir iii) to evaluate the spatial and temporal variability of sediment yield and identify the erosion prone area.

## 2 Materials and methods

### 2.1 Description of the study area

**Fig. 1:**
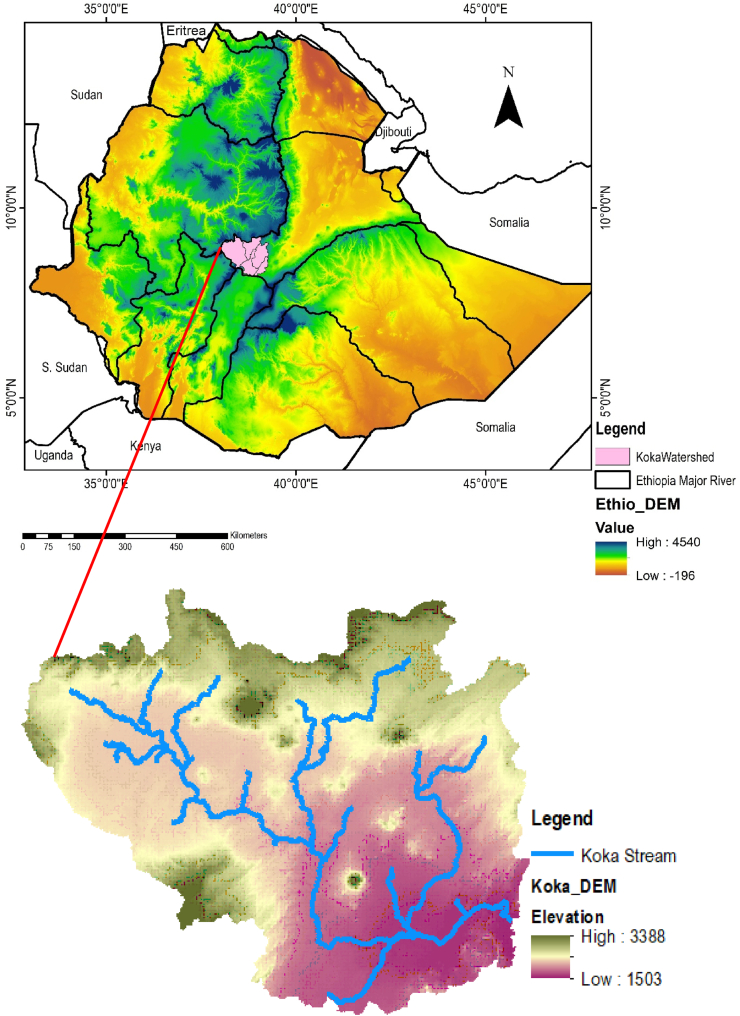
Location of the study area.

The Awash Basin is Ethiopia's second-largest basin, with a length of around 1200 km and a catchment area of 110,000 km^2^. It is located between 8^0^16^’^ and 9^0^18′ latitude and between 37^0^57^’^ and 39^0^17^’^ longitude with altitude varying between 215 and 4185 m a.m.s.l (above mean sea level) [46]. The study area is located on the upstream side of the Koka dam which covers 10,371.43 km^2^ watershed area with an altitude varying between 1503 and 3388 m. Koka Dam was built in 1960 for hydropower generation on the Awash River's upstream section, with a reservoir capacity of 1180 Mm^3^. The area is recognized as a prominent location where rapid population growth, agricultural practice, urbanization, and industry expansion occur, resulting in changes in the LULC from time to time. The Koka Dam watershed area is bounded to the south by the Rift Valley Basin, to the northwest by the Abbay Basin, to the west by the Omo-Gibe, and to the east by the Middle Awash. The detailed geographical location of the research area is presented below (Fig. 1).

The upstream of the Koka dam catchment got an annual mean rainfall of 1199.56 mm through the period of 2000–2015. The maximum, minimum, and average values of temperature observed at the watershed during the period 2000–2015 were 250C, 100C, and 17.50C respectively.

Commonly, the types of LULC in the UARB are Agricultural land (rainfed and irrigation crops), water bodies, forests, shrubs, woodland, and bare land. Crop land is the dominant LULC in the watershed. The principal crops grown in this region include teff, maize, barley, vegetables, wheat, beans, and sugarcane. In general, irrigation farms, sugar plants, wetlands, Addis Abeba (Ethiopia's capital city), and other small urban regions are found in the watershed. Because of fast population growth, human activity in this watershed is increasing, and natural resources are being depleted in unanticipated and unsuitable ways.

The most dominant soil groups in the catchment are vertosols and Cambisols. Calcic Fluvisols, Lithic Leptosols, Eutric Nitosols, Calcic Xerosols, and Vertic Cambisols are the most common types of soil in this watershed. The most common soil texture of the research area is sandy and silt clay loam.

### 2.2 Model input and data acquisition

The QSWATPLUS model uses spatial data (topography, DEM, soil, and land use maps) and meteorological data (precipitation, temperature, relative humidity, wind, and solar radiation). The hydrological data (streamflow and sediment concentration) were collected from the Minster of Water and Energy and used to calibrate and validate. The spatial, vector, climate, and hydrological data were collected from different sources to use in this study. The following are the model inputs that were used for the QSWATPLUS Model.

#### 2.2.1 Meteorological data

Thirteen meteorological observation stations were used where the stations located within and around the Koka Dam watershed. Climate data such as daily precipitation, temperature, wind, sunlight hour, and humidity were obtained from the Ethiopia National Meteorological Agency (ENMA) from 2000 to 2015.

**Fig. 2:**
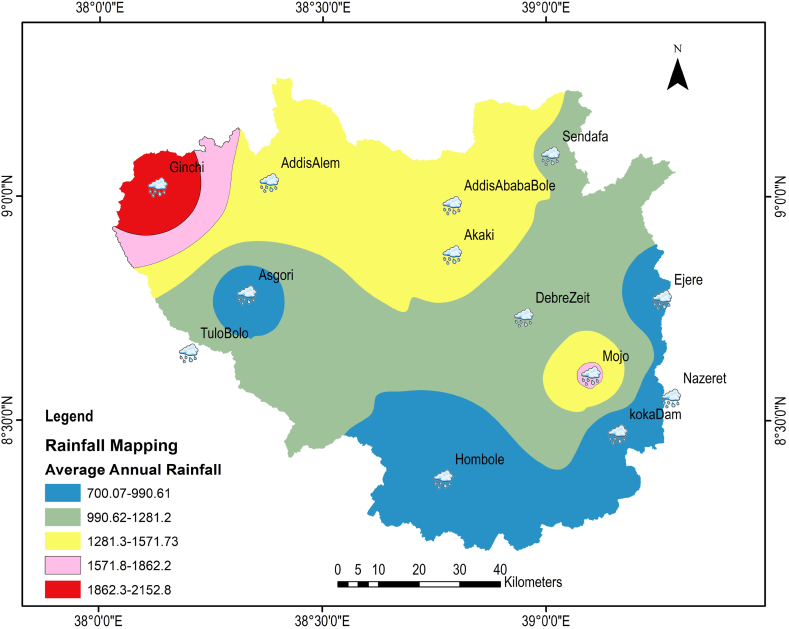
Average annual rainfall depth (2000–2015) of upper Awash metrological stations using inverse distance Weighted Interpolation Method.

The mean annual rainfall of Addis Ababa, Zazeret, KokaDam, Asgori, Hombole, Debrezeit, Mojo, Sendafa, Tulo Bolo, Addis Alem, Akaki, Ginchi, and Ejere meteorological stations for the period of 2000–2015 are shown below in Fig. 2.

#### 2.2.2 Observed streamflow and sediment data

The hydrological data such as daily observed streamflow and suspended sediment samples data were obtained from the Minster of Water and Energy to calibrate and validate the QSWATPLUS model at the Koka Dam gauging station. The calibration and validation were performed using 16 years of observed data (2000–2015). The sediment concentration data is a non-continuous time step that was collected from the Minster of Water and Energy. A sediment rating curve should be developed using observed sediment data as a function of the related stream flow data to obtain the continuous time-step sediment data [47].

The following equation can be used to express the relationship between streamflow and suspended sediment concentration:

(1) $$Q_{s}=a*{Qw}^{b}$$

(2) Qs=0.0864*Qw*C

Where C is the suspended sediment concentration (mg/lit), Qs is the suspended sediment concentration (ton/day), a and b are constants and Qw is the streamflow (m^3^/sec).

It is obtained from the empirical relationship between observed sediment data and associated streamflow data [48] and is equated as:

(3) $$\mathrm{Log}\left(Q_{S}\right)=a+b*\mathrm{Log}\;\left(Q_{W}\right)$$

The bias correction factor was used to improve the accuracy of the sediment rating curve, which calculates the sediment concentration load. The equation is as follows [49].

(4) $$\mathrm{Log}\left(Q_{S}\right)=a+b*\mathrm{Log}\;\left(Q_{W}\right)+\text{CF}$$

Based on Ferguson's (1986) study, a statistical bias correction factor is the exponent of (2.65 S^2^) which is used to minimize the error estimation using the sediment rating curve and the variance is equal to:

(5) $$S^{2}=\frac{\sum_{i=1}^{n}{\left\langle \mathrm{Log}\left(Ci\right)-\mathrm{Log}(Ĉi\right\rangle }^{2}\;}{n-2}$$

Where Ci is the observed value, Ĉi is the predicted value, and n is the total sample of data, S^2^ is the variance.

For this particular study, three rating curve development methods were used to derive the suspended sediment concentration rating curve using non-continuous time step sediment data and corresponding measured streamflow data such as normal linear log-log relationships, normal linear log-log relationships with a bias correction factor, and non-linear log-log relationships. The non-linear log-log relationship is calculated using the Microsoft Excel Solver Tool [50].

(6) $$\mathrm{Log}\left(Q_{S}\right)=a+b*\mathrm{Log}\;{\left(Q_{W}\right)}^{C}$$

Where a, and b are constants and c is the exponent coefficient.

**Fig. 3:**
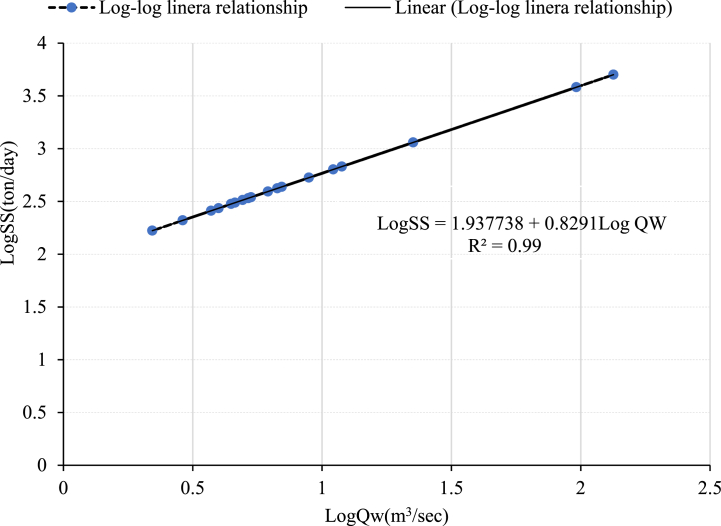
Log-Log linear relationship sediment rating curve developed at Koka dam station.

**Fig. 4:**
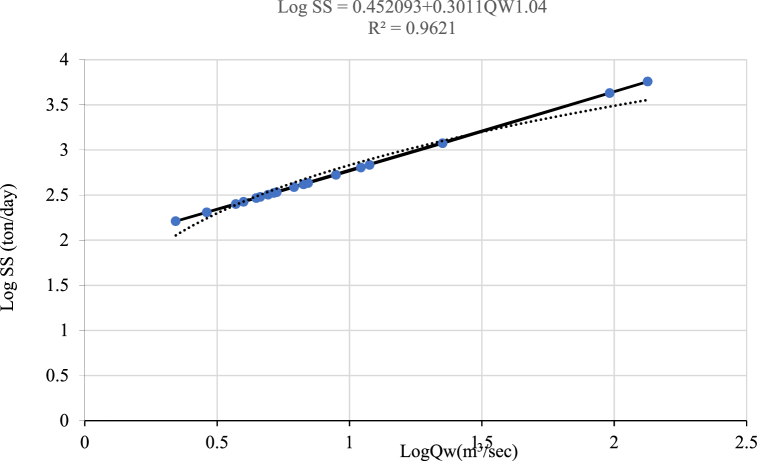
Log-Log nonlinear relationship sediment rating curve developed at Koka dam station.

For the Koka gauge and Hombole stations, a sediment rating curve is developed using the three equations mentioned above (Equation (3), (4), and (6)). Using goodness-of-fit, the optimal sediment rating curve was identified. The goodness of fit of the sediment rating curve is assessed using the coefficient of determination (R2), Nash-Sutcliffe efficiency (NSE), root mean square error (RMSE), observed standard deviation ratio (RSR), and percent bias (PBIAS). Based on the above statistical model performance indicator value the equation was selected [51]. The Koka dam gauging station's sediment rating curve results are shown in the charts below (Fig. 3, Fig. 4

#### 2.2.3 Spatial data

The spatial data including land sat images (remote sensing satellite), DEM, soil map, and vector data were obtained from the Earth Resources Observation and Science (EROS) data center of the United States Geological Survey (USGS) (http://espa.cr.usgs.gov) and the Ethiopia Geospatial Map Institute. Three historical satellite images were used to assess the impact of LULC change on watershed hydrology and sediment production, and a five-year gap was taken into account for the years 2005, 2010, and 2015. In addition, field observation and ground truth data were collected to validate the LULC classification.

#### 2.2.4 Landsat image data sources

Multi-variety satellite pictures in terms of spatial and temporal data were gathered to simulate and assess the effects of LULC change on sediment yield dynamics. The historical 2005,2010 and 2015 cloud-free satellite images were downloaded using Landsat-7 ETM+(Enhanced Thematic Mapper), and Landsat-8 OLI (Operational Land Imager) satellite. The landsat7 ETM + images (2005,2010) with Worldwide Reference System (WRS) Path 169 and Row 52 were acquired on May 20/2005, and March 2010 respectively, While the Landsat-8OLI satellite image was acquired on February 13/2015. The images of February, March, and May were used because of the best period of minimum cloud and sense cover images in Ethiopia which are the summer season starting from February up to May. The satellite image data have a 30 m resolution.

#### 2.2.5 Land use and land cover classification

The image processing such as layer stack, mosaic, merge, and clip image was performed using Quantum GIS and ERDAS-14 software. After image pre-processing, the satellite raster image is changed into a polygon shape using the signature editor then the LULC classification was done using the combination of the ERDAS supervise image technique and Google Earth. The supervised classification was performed using 536 training sampling locations obtained from georeferenced data and applied to the Landsat image using the maximum likelihood classification technique and the area covered by each LULC class. The supervised classification method outperforms other image classification methods currently in use. It is verified by using ground truth data.

#### 2.2.6 Accuracy assessment

The accuracy assessment was done using information which is collected from ground surveying data for each type of LULC. The survey data was collected using the Global Position System from February 1, 2022, to May 2022 time interval. Visual observations were made from 536 training locations where 110,40, 60,54,65,69,70, and 68 were taken from cultivated land, urban areas, bare land, shrubland, bushland, woodland, water bodies, and forest respectively. The majority of the observation data (384 sample points) were utilized for classification, while the remaining data (152) were used to evaluate the accuracy. Out of 384 sample training points, 78,28,42,38,46,50, 51, and 51 were taken from cultivated land, urban area, bare land, shrubland, bushland, woodland, water bodies, and forest, respectively. Besides collecting data, a discussion was made with community elders to check the similarity between the classification of the LULC type and the ground LULC type.

**Fig. 5:**
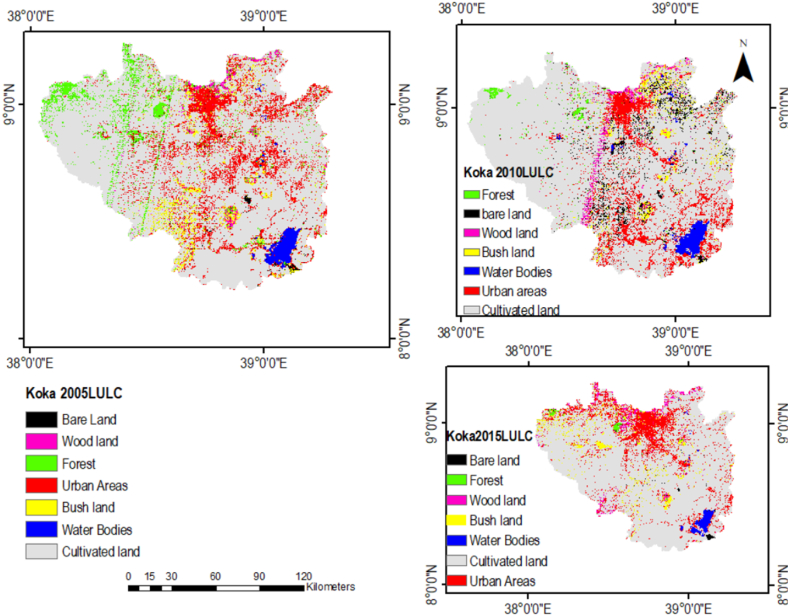
LULC classification 2005,2010, and 2015 LULC data using the supervised technique.

The accuracy assessment efficiency was calculated using the Kappa coefficient and the overall accuracy efficiency value is 0.81. Majorly, the LULC types in the Koka Dam watershed are cultivated land, woodland, shrubs, urban areas, bare ground, forest, woodland, and aquatic bodies (Fig. 5). During the classification of LULC, the following descriptions of LULC were considered (see Table 1).

**Table 1:** Classification of LULC change type from 2000 to 2015.

Classification of LULC type	Descriptions
Forest	Areas covered by natural high forests and large man-made trees

Woody land	Mainly the woodland falls next to the forest and is found in hill areas and along the riverside and it consists of medium bushes
Cultivate land	the area includes all agriculture practices like state farms and
	holder farms
Bushland	areas dominated by small bushes and shrub plants and
	sized plant species (less than 3 m)
Bare land	Areas of the exposed surface
Water Bodies	The water bodies include lakes, rivers, and other water bodies

### 2.3 QSWATPLUS model

SWAT+ is the most recently rebuilt version of the SWAT model and it is based on the concept of the hydrological response unit of watersheds. The hydrological response unit shows the catchment characteristics by identifying the soil type, land use type, and slope of watersheds.

Similar to the SWAT model, the SWATPlus model uses an equation to predict various aspects of the hydrological cycle, including surface runoff, infiltration, evapotranspiration, plant growth, sediment yield, routing, and many more. However, the SWATPlus model has significant advantages over the original SWAT model. It is suitable for large models and has virtually no size restriction. It is very flexible compared to SWAT. It also adds some features like landscape units. It is a semi-distributed hydrological model that simulates the response of the hydrology and the output of suspended sediment by using the dynamics of LULC [52]. The QSWATPlus model, which is also the most recent version, simulates surface runoff and sediment yield using the water balance equation [53].

$${SW}_{t}={SW}_{0}+\sum_{j=1}^{t}(R_{day}-Q_{surf}-E_{a}{-W}_{seep}-Q_{gw})\;(7)$$

Where SWt is the final depth of soil and water content (mm), SWo is the initial soil and water content on the time j (mm), t is the period (days), Rday is the measurement of the amount of rainfall on day j (in millimeters), Qsurf is the measurement of the amount of runoff on day j (in millimeters), Wseep is the measurement of the amount of water seeping into the soil layer on day j (in millimeters), Ea is the measurement of the amount of evapotranspiration on day j (in millimeters), and Qgw is the measurement of the return flow amount on the day (mm).

A modified universal soil loss equation (MUSLE) can be used to estimate sediment production:

(8) $$S_{ed}=11.8{\left(Q_{surf}*q_{peak}*{area}_{hru}\right)}^{0.56}*K_{USLE}*C_{USLE}*P_{USLE}*{LS}_{USLE}*CFGR$$

Where K_USLE_ is the soil erosion factor, C_USLE_ is crop management, P_USLE_ is plant management practice, LS_USLE_ is a topographic factor, CFRG is the coarse fragment factor, and Sed is the sediment yield from the catchment (tons). Qsurf is the amount of surface runoff volume (mm/ha), qpeak is the peak flow rate (m3/sec), and area_hru_ is the area of the hydrological response unit (ha).

### 2.4 Sensitivity analysis, model calibration, and validation

The model calibration process is complex and it needs iteration of the calibration process for all model parameters [54]. Under such cases, sensitivity analysis should be carried out to identify which parameters had a high impact on changing model results. Calibration took place between Jan 1/2000, and Dec 31/2010, and validation took place between Jan 1/2011, and Dec 31/2015. But the first year was taken as a warming-up period.

Both automatic and manual techniques were used to calibrate the simulated and measured values. By changing the parameter value, the corresponding model statical values were computed and the calibration continued until the acceptable model performance statical indicator value which was recommended by the QSWAT + developer. The recommended value for calibration of streamflow and sediment is coefficient determination (R^2^) >0.6 and Nash Sutcliffe Efficiency (ENS) > 0.5 [55].

Model validation is the testing of the capability of the model to simulate the variable without any calibration adjustment parameters at different periods and spaces [56].

### 2.5 Statically indicators for model performance evaluation

Model performance statical indicators are used to evaluate the model prediction capability. The performance indicator is important to examine the relationship between the simulation output and the measured value. The statical indicators are Nash Sutcliff Efficiency (NSE), coefficient of determination(R2), and percent bias. They were used to evaluate the capability of model simulation [57,58], and [53].

**Fig. 6:**
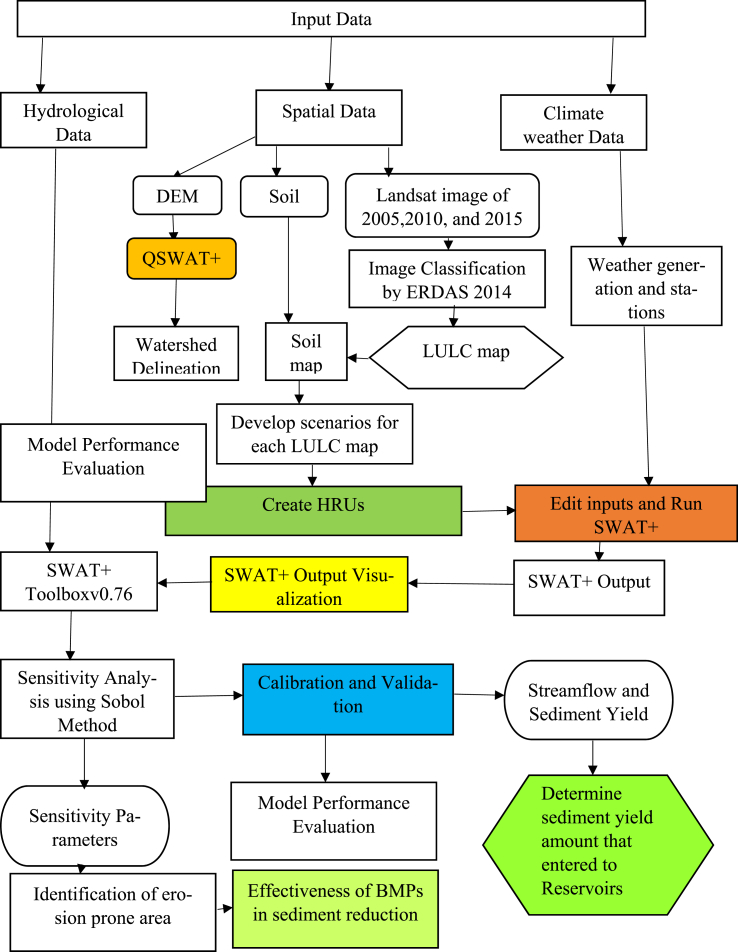
The overall methodology approach layout.

Generally, the overall methodology approach is shown below (Fig. 6).

### 2.6 Evaluation of land use change effect on sediment yield

The temporal and spatial effects of LULC alteration on sediment output were assessed using 2005,2010, and 2015 LULC scenarios. The temporal variance was analyzed using four time frames, namely 2000–2005, 2005–2010, 2010–2015, and 2000–2015, which reflect different LULC regimes in the watershed. Between 2000 and 2015, the spatial hydrological responses at the subbasin scale were evaluated.

### 2.7 Mapping erosion prone area and sediment reduction methods

Watershed management practices cannot be adopted over the entire watershed due to financial constraints, human resources, time constraints, and land availability in the targeted area. Identifying erosion-prone areas is essential for effective and efficient watershed management practices. In general, mapping the spatial distribution of sediment yield is critical for watershed management planning and strategies [13].

In this work, the best management practices adopted in various land use management scenarios were analyzed based on the efficiency of sediment reduction in the upper Awash basin. The development of BMPs in watersheds (critical subbasins) has been identified as an efficient technique to significantly reduce sediment erosion. The best management practices (BMP) scenarios were applied by adjusting QSWATPLUS parameters to understand the effects of practice on simulated results within the model. The selection of BMPs and the values of their parameters are site-specific and should represent the reality of the research area [59]. The best management strategies chosen are filter strips, terracing, and contour, which are utilized to manage sediment erosion areas in the upper Awash River basin. In this context, the best management practices were taken from a community-based Ethiopian water-shed management guideline and the selected best scenarios were similar to different findings involving catchments in Ethiopia [7,[60], [61], [62]], and [63]. The detailed discussion for each scenario is presented below.

**Baseline Scenario (1):** This scenario shows the existing condition of the watershed without any implementation of best management practices. In this simulation, the calibration and verification of the model were performed without changing any calibration parameters.

**Filter strips Scenario (2):** Different widths of filter strips were applied on all hydrological response units based on soil types, land use types, and slope classes. Because the filter strips are used to trap silt in a particular area. The filter width value was assigned based on local research experience in the Ethiopian highlands and the value of width is 1 m–30 m [64]. As a result, a 5 m wide filter strip is used for this study.

**Terrace Scenario (3):** The application of terracing in a watershed reduces the slope of the HR and the slope length of the subbasin. Terracing in QSWAT is simulated by adjusting both erosion and runoﬀ parameters. The simulation of the eﬀect of terracing on sediment reduction, USLE support practice factor (USLE-P), SCS curve number (CN2), and slope length of the hillside (SLSUBBSN) are the parameters to be adjusted based on the land slope. In general, parallel terraces with different slope lengths and stone bunds were placed on agricultural HRUs that are a combination of farmed land, all soil types, and slope classes. As a result, we chose a 30 % reduction in slope length for this investigation.

**Contour Scenario (4):** Contour planting parameters were modiﬁed in QSWAT by changing the curve number (CONT-CN) for surface storage and inﬁltration and the USLE practice factor (CONT_P) for erosion.

**Combination Scenario (5):** This scenario is implemented by adjusting the curve number, management practice, average sloping length, and basin slope.

## 3 Results

### 3.1 Land use and land cover change

Cultivate land was the most dominant LULC type in the Koka Dam watershed and it covered 75.51 % in 2000, 78.61 % in 2005, 81.66 % in 2010, and 82.82 % in 2015 (Table 2). In 2015, the urban area expanded by 6.34 %, and the coverage of cultivated or agricultural land increased by 7.31 %. Whereas, the forest land coverage decreased by 5.47 % in 2015. The significant expansion of cultivated land and urbanization areas have occurred in the upper Awash River basin since 1985. Based on Shawl and Chakma's study, the future LULC change scenarios of the years 2025 and 2035 will have a significant expansion of cropland and urban areas in the upper Awash River basin. They concluded that an expansive area covered in woodland and shrubland had been replaced by agricultural land and urban areas [15]. The LULC dynamics dramatically shifted from forest and shrubland to agricultural land, according to other studies [24,65]. The outcome is similar to those previous findings in the case of incremental agricultural land and urbanization area but the incremental rate is different from the previous finding. This study revealed that the forest area declined by 5.47 % between 2000 and 2015, although previous studies did not show the deforestation rate.

**Table 2:** LULC change in (2000–2015years).

Land use type	LULC (%)				Changes in LULC (%)		
	2000	2005	2010	2015	2000–2005	2005–2010	2010–2015
Cultivate land	75.51	78.61	81.66	82.82	3.1	3.05	1.16
Woodland	2.33	1.31	1.43	1.4	−1.02	0.12	−0.03
Urban Areas	5.4	9.9	10.52	11.74	4.5	0.62	1.22
Forest	5.92	4.92	4.77	0.45	−1	−0.15	−4.32
Water Body	2.65	1.67	1.89	1.31	−0.98	0.22	−0.58
Shrub land	4.32	3.28	2.57	3.36	−1.04	−0.71	0.79
Bare land	0.8	0.27	2.92	0.24	−0.53	2.65	−2.68

### 3.2 Sensitivity analysis

The SWAT Toolbox was used to conduct the sensitivity analysis. The parameters were ranked based on their impact on hydrological model simulation results. The most responsive parameters for flow simulation were curve number, saturated soil hydraulic conductivity, soil depth, available water capacity, and base flow recession coefficient. The most sensitive parameters for sediment yield simulation were soil erosion factor, sediment concentration for lateral, manning's “n” for overland, the parameter for sediment routing, and curve number. The sensitivity parameters were determined based on a high correlation between the ground observed data and the simulated value, as well as recommendations from several literature sources in the Awash River basin [24,66], and [19]. The sensitive parameters used to calibrate and validate streamflow and sediment yield were consistent with previous research in the Awash River basin [55,66]. The calibration flow and sediment parameters are shown below in Table 3.

**Table 3:** Sensitivity analysis for streamflow and sediment calibration.

List Parameters	Description	Ranks		Parameters range
		Flow	Sediment	
ALPHA_BF	Base flow recession coefficient	5		0.0–1.0
CN2	Initial curve number	1	5	35–95
CH_K	Channel hydraulic conductivity	6	6	0.0–150.0
ESCO	Soil evaporation compensation factor	7	7	0.0–1.00
Soil_Z	Soil depth	3		0.0–3500.0
Soil_k	Saturate soil hydraulic permeability	2	8	0.0–2000.0
SOL_AWC	Soil storage capacity or content	4		0.0–1.00
SLOPE	Slope rank		9	0.0–0.9
SPCON	Parameter for sediment routing		4	0.0001–0.01
USLE_P	Cover factor	8	10	0.1–1.0
REVAP_MIN	Percolation to the deep aquifer t occur	9		0.0–500
OVN	Manning's value for Overland	10	3	0.01–30
USLE_K	Soil loss factor		1	0.0–0.65
LAT_SED	Lateral sediment concentration		2	0.0–5000

### 3.3 Calibration and validation

**Fig. 7:**
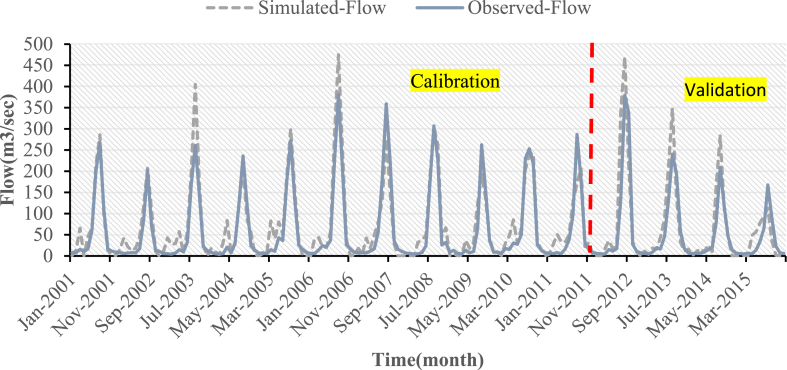
Streamflow calibration and validation at Koka dam stations for 2005 LULC.

**Fig. 8:**
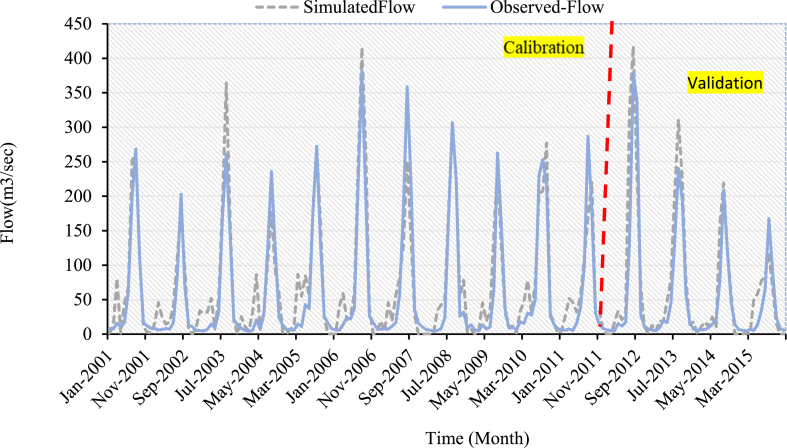
Streamflow calibration and validation at Koka dam stations for 2010 LULC.

**Fig. 9:**
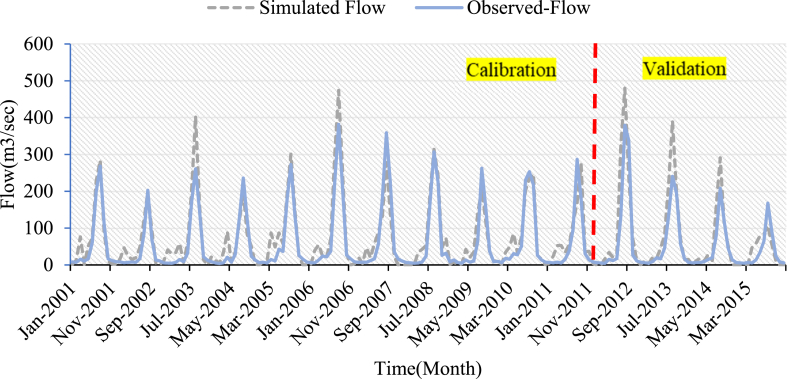
Streamflow calibration and validation at Koka dam stations for 2015 LULC.

**Fig. 10:**
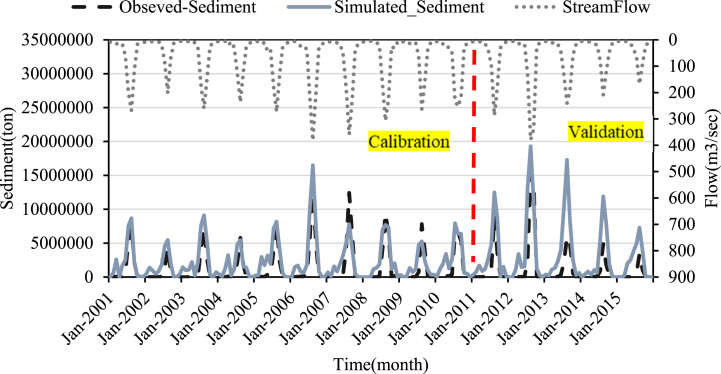
Sediment calibration and validation at Koka dam stations for 2005 LULC.

**Fig. 11:**
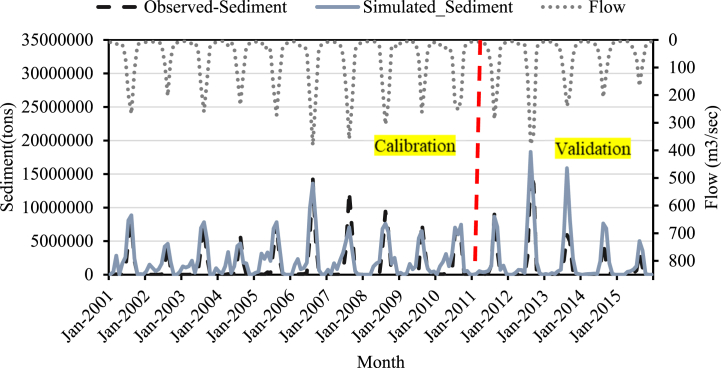
Sediment calibration and validation at Koka dam stations for 2010 LULC.

**Fig. 12:**
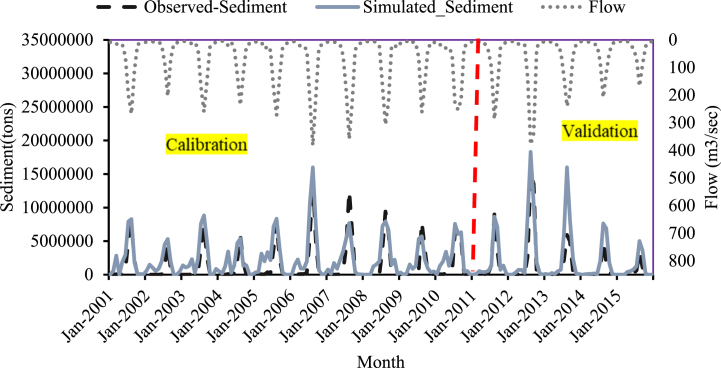
Sediment calibration and validation at Koka dam stations for 2015 LULC.

Calibration and validation were performed using monthly measured flow and sediment yield data at the outlet of the Koka Dam watershed. The simulated values of streamflow and sediment yield for the three LULC scenarios have a good match to the ground observed values, as shown in Fig. 7, Fig. 8, Fig. 9, Fig. 10, Fig. 11, Fig. 12. However, the streamflow and sediment yield simulations are higher than the actual values. This demonstrated that the model overpredicted the streamflow and sediment. This could be due to data quality and lack of sediment data. The hydrograph of streamflow which is indicated in Fig. 7 up to **9** showed that the simulated flow and observed flow for the 2005 LULC scenario had a better relationship when compared to 2010 LULC and 2015 LULC map results. The statical values of (R^2^) for the 2005 LULC, 2010 LULC, and 2015 LULC data were 0.91, 0.89, 0.89, and 0.77,0.81,0.79 during calibration and validation, respectively (Table 4). This result showed that the model efficiency was at good performance. This ensured a high correlation between the predicted and observed flow. The model performance results for this study were consistent with findings from prior research projects carried out in the upper Awash River basin [25].

**Table 4:** Model performance result for streamflow calibration and validation at Koka dam.

Model Performance Indicators	2005 Historical LULC		2010 Historical LULC		2015 Historical LULC	
	Calibration (2000–2010)	Validation (2011–2015)	Calibration (2000–2010)	Validation (2011–2015)	Calibration (2000–2010)	Validation (2011–2015)
R2	0.91	0.77	0.89	0.81	0.89	0.79
NSE	0.89	0.7	0.88	0.77	0.87	0.67
Percent Bias	−16.70	−18.11	−14.86	−21.12	−12.74	−28.77

The simulated and observed monthly flow at the Koka Dam watershed outlet was plotted for visual comparison Fig. 7, Fig. 8, Fig. 9. The hydrograph of the calibration and validation period of observed and simulated streamflow showed the model slightly overestimated the peak value during the rainy season. As displayed in Fig. 7, Fig. 8, Fig. 9, the simulated peak flow during the rainy season exceeded those measured values in three LULC scenarios. The average annual measured stream flow was 59.99m3/sec, whereas the mean annual simulated stream flow was 70.3 m^3^/s, 72.3 m^3^/s, and 73.5 m^3^/s for the 2005, 2010, and 2015 LULC data, respectively. This demonstrated that the change in LULC was the reason for the increase in mean annual stream flow, which increased from 70.3 m^3^/s to 73.5 m^3^/s between the years 2005 and 2015.

The monthly time-step sediment yield hydrograph was developed to show the simulated and actual sediment load values (2000–2015) during the calibration period. Fig. 10, Fig. 11, Fig. 12 depict the sediment yield hydrograph for measured and simulated values between 2000 and 2015.

The result of calibration and validation revealed an excellent correlation between simulated and observed sediment values. The model performance result also showed a satisfactory result. As shown in Fig. 10, Fig. 11, Fig. 12 the simulated sediment value exceeds the observed sediment values. This showed that the model overpredicted the sediment value. R^2^, NSE and percent bias during calibration for the 2005 LULC data were 0.81, 0.68, and −66.18, respectively (Table 5). The model performance result is better than the previous study results in this area [25].

**Table 5:** Model efficiency for sediment calibration and validation at Koka Dam gauge station.

Model Performance Indicators	2005 Historical LULC		2010 Historical LULC		2015 Historical LULC	
	Calibration (2000–2010)	Validation (2011–2015)	Calibration (2000–2010)	Validation (2011–2015)	Calibration (2000–2010)	Validation (2011–2015)
R^2^	0.81	0.71	0.78	0.79	0.78	0.68
NSE	0.68	0.61	0.66	0.6	0.64	0.6
Percent Bias	−66.5	−75.5	−64.23	−53.5	−68.9	−54.6

### 3.4 Temporal variability of sediment yield, evaporation, and surface runoff

**Fig. 13:**
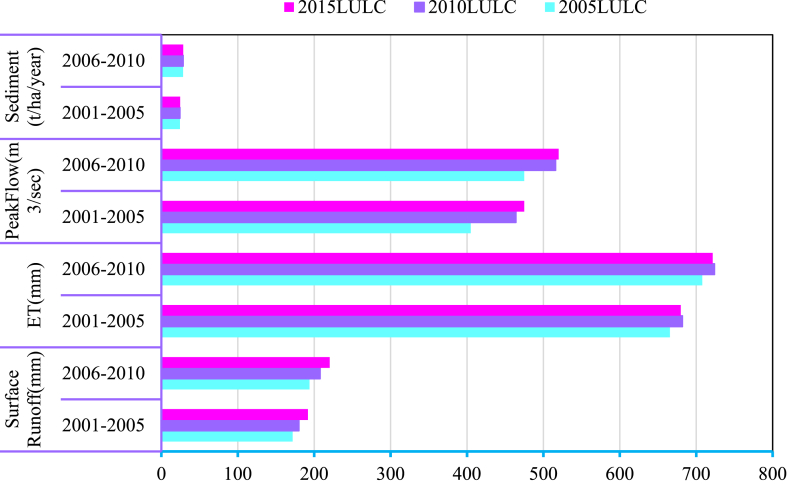
Temporally variations of hydrological response under land use change.

**Fig. 14:**
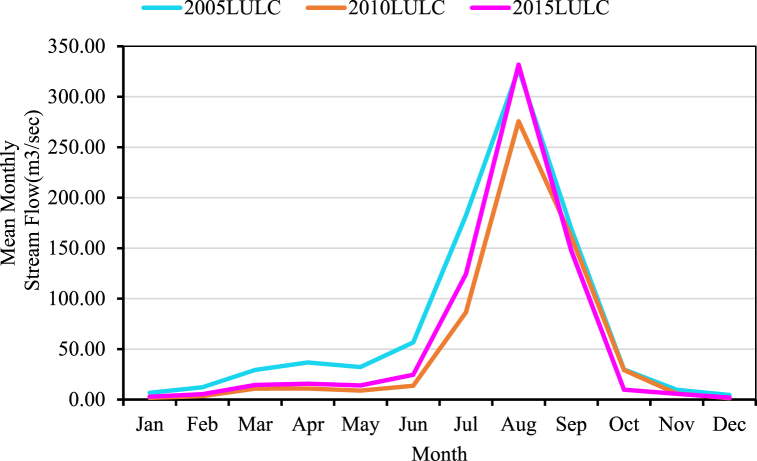
Monthly average streamflow comparison under three land use references data (2001–2015).

The three LULC scenarios were used to evaluate the temporal effects of LULC changes on surface runoff, evaporation, peak flow, and sediment yield. As shown in Fig. 13, the amount of surface runoff volume, peak flow, and evaporation during the period between 2006 and 2010 is greater than the values of the period between 2000 and 2005. The hydrological responses with different periods of LULC change scenarios are prepared and shown in Fig. 14. The mean annual streamflow and sediment yield of the Koka Dam watershed were increased year to year due to LULC effects. This was due to an increase in agricultural practice areas. The Koka Dam watershed changed from forest plantations to agricultural land between 2000 and 2015 periods. As a result, the runoff and sediment yield increased by 12.68 % and 8.84 % respectively. These findings are similar to other studies conducted in different catchments. For example, M.choto et al. (2019) described the sediment yield of the Gojeb watershed increasing over time due to an increase in agricultural land area [67]. Similarly, the surface runoff of the Andassa watershed increased due to changes in land use and land cover [68].

### 3.5 Spatial variability of sediment yield, surface runoff, and evaporation at subbasin scale

The QSWAT-Plus model was used to evaluate the impact of LULC changes on hydrological responses at the subbasin level using 2005,2010,2015 LULC data. The hydrological responses vary from subbasin to subbasin due to land use/cover change, DEM, soil type, rainfall distribution, slope classes, and management practice. Subbasin 2 has the largest change in surface runoff, water yield, and evaporation when compared to the other subbasins (Table 6).

**Table 6:** The spatial variation of surface runoff due to LULC change.

Subbasin	2005LULC			2010LULC			2015LULC		
	Surface Runoff(mm)	Water Yield(mm)	Evaporation(mm)	Surface Runoff(mm)	Water Yield(mm)	Evaporation(mm)	Surface Runoff(mm)	Water Yield(mm)	Evaporation(mm)
1	166.58	224.96	736.59	163.46	196.13	756.68	177.84	204.66	753.48
2	281.16	357.56	765.37	283.46	333.98	768.55	314.84	358.01	762.81
3	202.58	237.98	668.63	199.33	224.12	686.29	215.24	241.04	720.64
4	117.26	129.72	554.37	133.60	142.99	561.63	135.53	143.69	561.30
5	162.71	177.16	634.53	176.58	185.36	627.71	214.16	221.48	623.99

### 3.6 Spatial mapping of sediment yield at subbasin scale

**Fig. 15:**
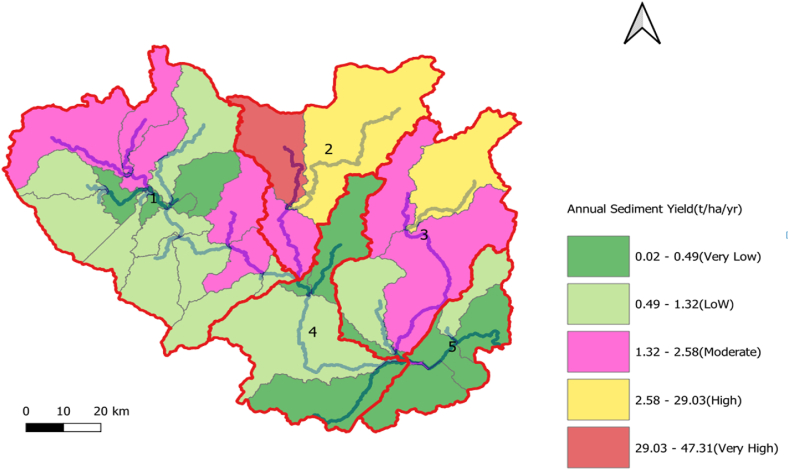
Spatial mapping of mean annual sediment yield using 2015 LULC (Base-line).

The spatial mapping of sediment yield over the entire watershed is displayed in Fig. 15. The simulated mean annual sediment yield of the watershed ranged from 0.02 to 47.31 t/ha/yr, with a mean value of 28.33 t/ha/yr in the baseline scenario. Fig. 15 indicates that subbasin 2 was the most erosion-prone area with an average value of 29.03–47.31 t/ha/yr. In the Koka Dam watershed, 18.93 % of the areas are classified as very high erosion-prone, 28.4 % and 35.1 % as high and moderate erosion-prone, 11.5 % as lowly prone to erosion, and 7.8 % as very low prone to erosion.

Subbasin 2 is dominantly covered by agricultural and urban land which caused to increased erosion rate. Agricultural practice contributed the most sediment yield in subbasin two. Based on Estifano's reported sediment yield was increased due to an increase in agricultural land and a decrease in forest land [69]. Another study also described that cultivated or agricultural land contributed more sediment yield to the Megech reservoir [47].

### 3.7 Identifying best management practices (BMPs)

**Fig. 16:**
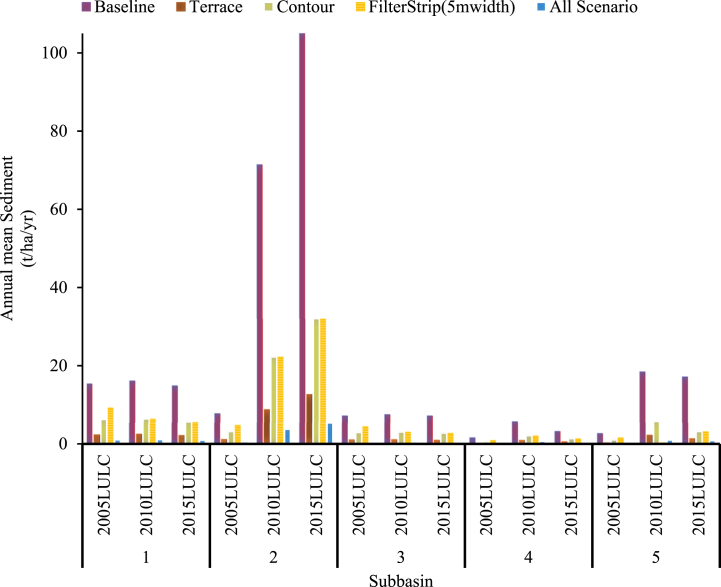
Mean annual simulated sediment yield for each scenario.

It was identified that subbasins 2 and 3 are categorized as high erosion-prone areas. These subbasins contribute to the Koka Dam reservoir with an average annual sediment yield of ≥29.031 t/ha/year. As a result, this watershed underwent the implementation of three chosen management approaches. As shown in Fig. 16 terrace was the best sediment reduction scenario method among those selected methods which was tested in three LULC scenarios. This finding was differed from other studies in this study area. For instance, N. Boru et al., study (2022) found that terraces, grass waterways, and filter strips were the best management practices and the sediment reduction scenario was done using one specific year of LULC data. However, in this particular study, the sediment reduction method was evaluated using three LULC scenarios. According to their findings, the terrace application reduced the amount of sediment yield compared to the grass waterway and filter strip. This finding also indicated that the terrace had better sediment reduction than the contour and filter strip. A similar discovery has been made in the Kesem Dam watershed [70]. They found that applying terracing in watershed management was the best option to reduce sediment that would enter into reservoirs.

## 4 Discussion

### 4.1 Impact of land use and land cover change on streamflow

The impact of LULC change in the Koka Dam watershed significantly affected the hydrological response. For instance, the average annual surface runoff for the recent 2015 land use/cover was increased by 12.65 % from the 2005 historical land cover. The mean annual streamflow of the Awash River at the outlet of the Koka Dam watershed was increased by 3.2 m^3^/s within 16 years. These changes in streamflow were due to the majority area of forest and woodland being converted to urban areas and agricultural land. The increase in surface runoff is primarily caused by a decrease in the infiltration rate when forest land is converted to other land uses. In general, surface runoff has increased in the study area within 16 years. These findings support previous studies that found surface runoff increased as a result of LULC changes [65]. Furthermore, these results are comparable to those of other research carried out in various catchments. For example, Kuma et al., 2023 reported an increase in surface runoff in the Bilate catchment as a result of LULC alterations [20]. Similarly, changes in LULC in the Megech Dam watershed were found to increase surface runoff [47].

### 4.2 Effect of land use and land cover change on sediment yield

The effects of LULC changes on sediment yield dynamics in the Koka dam reservoir are higher when compared from time to time. Because the LULC drastically changed from forest and shrubland to agricultural land and urban areas. The amount of mean annual sediment load entering the Koka Dam reservoir was 26.03 t/ha/year in 2005 and increased to 26.34 t/ha/year in 2010 and 28.33 t/ha/year in 2015. Due to the woodland and forest land converted to agricultural land, the contribution of mean annual sediment yield increased by 2.3 t/ha/year from 2000 to 2015. Moreover, over 16 years period (2000–2015), there was an increase of cultivated land area by 7.31 % causing an increase of sediment yield by 47.31 t/km^2^/year in the study area. This led to increased soil erosion in this watershed and increased sediment production in the reservoirs of the Koka dam. As a result, the capacity of hydropower generation of the reservoir has decreased from the expected design capacity. Similar results have been noted in this research region; the Koka reservoir's silt accumulation reached 481 million meters, resulting in an estimated 60 million Ethiopian Birr in economic losses and 128 million kWh of energy loss [9]. Similar findings have also been reported in other studies conducted in different parts of Ethiopia. For instance, Boru et al. (2022) [25] reported that the average annual sediment yield of the Melka Werer watershed is 21.43 t/ha/year. According to Kidane et al. (2019) [71], the mean annual soil erosion rate of the Guder watershed ranges between 25 and 30 t/ha/year.

### 4.3 Temporal land use effects on streamflow and sediment yield

The amount of surface runoff, evapotranspiration, peak flow, and sediment yield varied throughout the time as LULC altered. The simulated hydrological responses using the three LULC scenario data were divided into two time frames (2001–2005), and (2006–2010) to understand the effect of LULC change over time series. The periods from 2006 to 2010 showed the maximum variation in hydrological responses, while the periods from 2001 to 2005 showed the minimum variation. The maximum variation of hydrological response occurred between the 2005 LULC and 2015 LULC models which the surface runoff volume and peak flow rate values were 48.6 mm and 115 m^3^/s, respectively. Surface runoff, peak flow, and sediment yield values in the 2015 LULC model are higher than those in the 2010 LULC and 2005 LULC models. Every year, the sediment yield increased during the month of high precipitation occurrences. About 60.8 % of the total annual sediment yield occurred during the rainy season. It implies that the surface runoff is greater in the months of July, August, and September [72]. Similar research revealed that July, August, and September recorded 70.8 % of the overall sediment production [27].

### 4.4 Sediment yields spatial variability

The sediment variability of sediment yield for each subbasin in the watershed was identified using three LULC scenarios, and the simulated values range from 0.02 to 47.03 t/ha/yr. Sediment yields from each subbasin varied due to the combined effects of LULC, soil type, slope, weather, and runoff conditions. The areas of high sediment yield are located in cultivated land, shrub/bushland, urban areas, and bare land with steep slopes. Consequently, the annual sediment yield rate from each subbasin region was used to obtain a map of the spatial variability of sediment yield in the watershed, which showed the subbasins with the worst erosion severity classes. The severity of soil erosion in the watershed points to the need for careful control of sediment reduction in the subbasins especially susceptible sections, which was also concluded by the authors of [25].

According to Hurni's (1985) study, the tolerable soil loss rate in different agroecological zones of Ethiopia is estimated to be between 2 and 18 t/ha/y. However, subbasins 2 and 3 showed that annual mean sediment yields significantly exceeded the allowable rate of soil loss. Maximum sediment yields were recorded in subbasins 2 and 3, with an amount of 105.1 and 71.8 t/ha/yr, respectively. These outcomes are obtained under the 2015 LULC scenario. The main sources of high sediment yield are agricultural land, bare land, shrubland, and steep slopes. This demonstrates that the regional variance in sediment yield can be linked to the type of land use, soil, and slope classes. A similar study conducted in the Kesem dam watershed indicated that shrubland, agricultural, forest, bare land, and pasture land covers contributed a significant amount of sediment yield in catchments and slope had an impact of the highest sediment yield in the subbasins [19]. Therefore, the spatial variability of sediment output in the catchment and subbasin is crucial for the implementation of optimum management techniques of soil erosion in the upper Awash River basin.

### 4.5 Effective management options on soil loss

It was identified that subbasins 2 and 3 were classified as high erosion source areas. The subbasins contribute an annual average sediment yield of ≥28.33 t/ha/year. After identifying the hotspots in subbasins, BMPs will be implemented to reduce sediment output from the watershed. Several factors such as the source of sediment, the cost implementation, sustainability, the efficiency of BMPs in reducing sediment yield, the amount of rainfall, land use, and the slope of the sub-basins should be considered while selecting appropriate management practices. Consequently, in this study, inexpensive and more effective BMPs for reducing sediment yield were applied to those sub-basins greater than 28.33 tons/ha/year. The comparison was done using three LULC scenarios.

Following the establishment of terracing in the UARB, the mean annual sediment yields of AWRB in 2005 LULC, 2010 LULC, and 2015 LULC data were 1.87, 5.37, and 6.1 t/ha/yr, respectively. In this scenario, the mean annual sediment yield of the catchment reduced by 84 %,86.5 %, and 87.5 % in 2005,2010 and 2015 LULC data, respectively. Terraces can substantially reduce sediment yield, especially during high rainfall events and natural hillslopes. Similar findings have been observed in the Upper and Middle Awash River Basin (UAMRB) and Kesem Dam watershed. According to the author [25], the use of terracing resulted in a reduction of sediment yield of 19.56 % from the baseline scenario. They conclude that the area covered by agriculture and bare land achieved the maximum silt decrease.

Implementing a 5 m filter strip reduces sediment yield by 40 %, 71 %, and 69.6 % for the 2005, 2010, and 2015 LULC scenarios, with mean annual sediment yields of 7.01, 11.43, and 14.98 t/ha/yr, respectively. When the 10 m filter strip was used, the sediment was reduced by 60 %, 81 %, and 80 % for the 2005,2010, and 2015 LULC scenarios. These findings suggest that widening the filter strip can improve the reduction efficiency of sediment yield-prone subbasins. Since the effectiveness of filter strips increases as the slope of a field is kept less than 20 %, the simulation result for this scenario was found to be higher than the simulation result reported by Sisay. This study's findings are consistent with prior findings in Ethiopia [19,73].

The simulation result of contour reduced the average annual sediment output for the 2005 LULC,2010 LULC, and 2015 LULC data by 60 %,66.8 %, and 69.6 % from the baseline simulation of 11.69,39.87, and 47.03 t/ha/yr, respectively.

During the combination of filter strips, Contour, and terrace scenarios, the amount of sediment yield in UARB reduced by 93.3 %, 94.2 %, and 94.6 % for the 2005,2010 and 2015 LULC data, respectively. The average annual sediment yield of the watershed for the 2005,2010 and 2015 LULC data was 11.69,39.87, and 47.31 t/ha/yr, respectively.

### 4.6 Limitation of study

This study helps to understand the impact of LULC changes on sediment yield dynamics in the Koka Dam watershed using three LULC scenarios. However, other important physical variables such as phosphorous and nitrates were not considered in this study due to lack of reliable data. In addition to this, this study showed the efﬁciency of BMP implementation in reducing sediment yield, but the economic feasibility of these implementations was not evaluated due to the absence of data.

## 5 Conclusion

Changes in land use and land cover are key sources of soil erosion at the watershed, basin, regional, and global levels. The QSWAT Plus model was used to assess the effects of LULC changes on sediment yield dynamics in the Koka Dam watershed. The analysis of LULC change between the three references LULC (2005,2010, and 2015) revealed that over the past 16 years, LULC change had a significant impact on the upper Koka Dam watershed. Over 16 years, these were showed by increases of 7.31 % and 6.34 % in agricultural land and urban area, respectively. In contrast, the coverage of plantation forests, water bodies, and woodlands decreased by 5.47 %, 5.7 %, and 3 %, respectively. Runoff and sediment yield variables significantly changed when the Upper Koka Dam watershed's LULC map was changed keeping the catchment variables remained constant. Through sixteen years, streamflow, surface runoff, and sediment yield have all increased by 4.55 %, 12.68 %, and 8.84 % respectively. The simulated mean annual sediment yields of the Koka Dam watershed for the three LULC scenarios (2005,2010,2015) were 26.03, 26.34, and 28.33 t/ha/yr respectively. Some subbasins produced larger sediment output, ranging from 71.8 to 105.1 t/ha/yr. Therefore, identifying and mapping the spatial distribution of sediment yield is critical to minimize the erosion rate. Three management scenarios (filter strip, contour, and terraces) are used to reduce sediment yield. Filter strip, contour, and terrace reduced sediment yield by 60 %, 65 %, and 86 %, respectively, when compared to the baseline situation. By the overall, combination of filter strip, contour, and terrace, the amount of sediment yield in UARB was reduced by 94 %. Based on the research findings, it is recommended that the terrace scenario be implemented on the upper side of the Koka Dam watershed for efficient sediment reduction. Areas like steep slopes and extensive agricultural practices have a high risk of erosion categorized as high and very high. The differences in erosion risk among sub-basins help planners identify and prioritize certain catchment areas that require immediate soil conservation measures. The modeling technique may be useful to decision-makers in detecting potential soil erosion causes and identifying hotspot areas. The findings of the study will help policymakers in making watershed management decisions and can be used as a model for land use implementation in other watersheds. The simulated effective BMPs can be utilized to prevent soil erosion in the Ethiopian highlands and other similar watershed zones across the world.

## Funding statement

This research did not receive money from any financial grant agencies like private, governmental, and commercial or non-profit sectors.

## Data availability statement

Data will be made available on request.

## CRediT authorship contribution statement

**Bayu Geta Bihonegn:** Writing – original draft. **Admasu Gebeyehu Awoke:** Supervision.

## Declaration of competing interest

The authors declare that they have no known competing financial interests or personal relationships that could have appeared to influence the work reported in this paper.
